# Tyrosine Kinase Inhibitors-Induced Arrhythmias: From Molecular Mechanisms, Pharmacokinetics to Therapeutic Strategies

**DOI:** 10.3389/fcvm.2021.758010

**Published:** 2021-11-19

**Authors:** Mengfei Cheng, Fang Yang, Jiahui Liu, Dan Yang, Shuo Zhang, Yang Yu, Shuai Jiang, Mei Dong

**Affiliations:** ^1^Department of Pharmacy, Harbin Medical University Cancer Hospital, Harbin, China; ^2^The First Department of Respiratory Medical Oncology, Harbin Medical University Cancer Hospital, Harbin, China; ^3^Department of Clinical Laboratory, Harbin Medical University Cancer Hospital, Harbin, China

**Keywords:** TKIs, QT prolongation, atrial fibrillation, molecule mechanism, therapeutic strategies, pharmacokinetics

## Abstract

With the development of anti-tumor drugs, tyrosine kinase inhibitors (TKIs) are an indispensable part of targeted therapy. They can be superior to traditional chemotherapeutic drugs in selectivity, safety, and efficacy. However, they have been found to be associated with serious adverse effects in use, such as myocardial infarction, fluid retention, hypertension, and rash. Although TKIs induced arrhythmia with a lower incidence than other cardiovascular diseases, much clinical evidence indicated that adequate attention and management should be provided to patients. This review focuses on QT interval prolongation and atrial fibrillation (AF) which are conveniently monitored in clinical practice. We collected data about TKIs, and analyzed the molecule mechanism, discussed the actual clinical evidence and drug-drug interaction, and provided countermeasures to QT interval prolongation and AF. We also pooled data to show that both QT prolongation and AF are related to their multi-target effects. Furthermore, more than 30 TKIs were approved by the FDA, but most of the novel drugs had a small sample size in the preclinical trial and risk/benefit assessments were not perfect, which led to a suspension after listing, like nilotinib. Similarly, vandetanib exhibits the most significant QT prolongation and ibrutinib exhibits the highest incidence in AF, but does not receive enough attention during treatment.

## Introduction

Protein kinases are enzymes that catalyze adenosine triphosphate (ATP) γ-phosphate transfer to tyrosine residues of the substrate protein and regulate many essential cellular biochemical functions including differentiation, proliferation, and death (1). More than 500 kinases have been discovered, and depending on their substrate specificity, they can be divided into two categories: catalytic tyrosine/tyrosine-like phosphorylation and serine/threonine phosphorylation (2). More than 30% of proteins may be modified by the kinases in the human body (3) and over 50% of proto-oncogene and oncogene products have protein tyrosine kinase activity (4). In this way, tyrosine kinase inhibitors (TKIs) by competitive inhibition of the ATP binding pocket, inhibit tumor proliferation, which has been widely used in cancer target therapy (1, 5).

TKIs can be divided into monoclonal antibody drugs and small molecule TKIs. Monoclonal antibody drugs are mainly bound to unique epitopes on the extracellular matrix to regulate downstream signal transduction, thereby inhibiting the proliferation, invasion, and angiogenesis of tumor cells. Small molecule TKIs act by intracellular inhibition of phosphorylation (6). So, inherently, small molecule TKIs are less selective than monoclonal antibodies and may lead to more adverse effects (AEs).

After the first TKI drug imatinib was approved in the US in 2001, a total of more than 30 TKIs were approved by the FDA up to 2020 (7). Although all the approved TKIs can inhibit BCR-ABL1, they still have different targeted sites and distinctive potency and activity. First-generation TKIs, like imatinib, dramatically improved the 5-year survival from 11 to 90% in Philadelphia chromosome-positive chronic myeloid leukemia (CML) patients (8). Second-generation TKIs, such as nilotinib (9) and dasatinib (10), exhibited the ability to overcome imatinib resistance and a more rapid molecule response. Third-generation TKI ponatinib is the only drug that works against BCR-ABL1^T315I^ mutation (11).

As successful as TKIs are, they still face some challenges, such as AEs caused by drug poor selectivity, drug resistance, and other reasons. According to the FDA's Adverse Effects Reporting System (FARES) database's cardiovascular (CV) toxicity section, TKIs were considered to be the suspected drug in 83.2% of CV events. And torsade de pointes/QT prolongation was considered the only acute event, which had a 6.8% incidence, higher than other anticancer drugs (1.4%) (12). Similarly, correlations were found between QT prolongation with increased risk of polymorphic ventricular tachycardia, which leads to lethal arrhythmia and subsequent sudden cardiac death (SCD) (3, 7, 12). The mechanisms and countermeasures of TKI-induced arrhythmia are still unknown. To this purpose, we wrote this paper to provide a broad overview for the potential of approved TKIs in prolonging QT interval and atrial toxicity and systemic and comprehensive treatment for patients.

**Figure 1:**
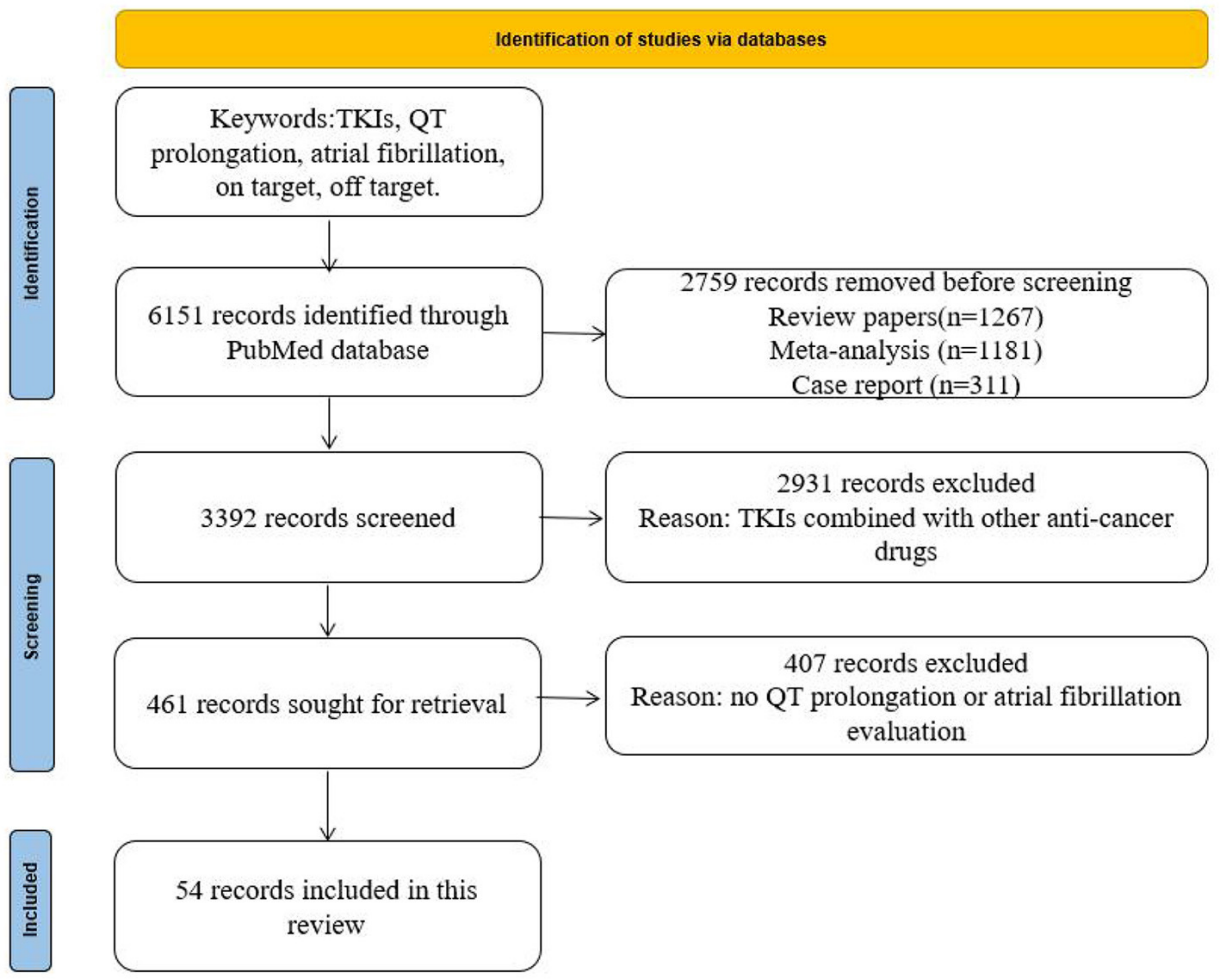
Search strategy and selection criteria.

As shown in Figure 1, the data for this review were identified by searches of PubMed and references from relevant articles using the search terms “TKIs,” “QT prolongation,” “atrial fibrillation,” and “on and off-target.” We identified 6,151 records through the PubMed database. We removed 2,759 records as they were review papers (*n* = 1,267), meta-analyses (*n* = 1,181), or case reports (*n* = 311). A total of 2,931 studies on TKIs combined with other anticancer drugs were also removed. Then, 407 records were excluded by reading the abstract. Finally, 54 records were enrolled. Only articles published in English between 2005 and 2021 were included.

## Potential Molecule Mechanism of QT Prolongation and Atrial Toxicity

Most TKIs are multi-target drugs, and the target receptors include vascular endothelial growth factor receptor (VEGFR), BCR-ABL, platelet-derived growth factor receptor (PDGFR), epidermal growth factor receptor (EGFR), and c-KIT, etc. (13, 14). Then they regulate the downstream signaling pathways, for example, PI3K, MEK, and AKT, etc. (14, 15). Only a few TKIs have only one or two targets, such as axitinib, bosutinib, and gefitinib (3). Due to the numerous targets of TKIs, the potential mechanisms of TKI-related side effects previously proposed can be divided into: “on-target” and “off-target” (14, 16) effects.

### On-Target

On-target means that the targets of TKIs exist in the tumor cell, but also play an important role in other normal organ cells, which may damage the biochemical function of normal cells (17, 18). A typical example of an on-target effect was observed in the Lu et al. trial. They designed an experiment to expose canine ventricular myocytes to drugs that have been demonstrated to prolong QT interval. As the result showed, the inhibition of the PI3K signaling pathway was the actual reason for QT prolongation. After blocking PI3K signaling, with the increase of persistent sodium current (*I*_NaP_) and the decrease of L-type calcium current (*I*_CaL_) and potassium current (*I*_Kr_ and *I*_Ks_), the total sodium and potassium current change accounted for over 70% of the whole prolongation, not just the potassium channels. And then, PI3K and its second message manager phosphatidylinositol 3,4,5-trisphosphate (PIP3) could affect multiple ion channels, similarly, resulting in action potential duration (APD). They also confirmed this result by breeding mice with reduced PI3K signaling showed QT prolongation (19).

Then, in McMullen's trial, they found that mice with decreased activity of the PI3K-Akt pathway was associated with higher susceptibility to AF and the same observation was relevant in humans. This pathway is an important regulator of cardiac protection under stress conditions. Other experiments also made a hypothesis that AF was related to ROS signaling, which would occur in abnormal Ca^2+^ release and atrial remodeling (20).

### Off-Target

Off-target refers to a non-selective TKI acting on normal organ cells. However, the target does not exist in tumor cells (21, 22). Generally, whether a TKI drug has the effect of QT prolongation, we can first observe whether there are fluorophenyl or fluoromethyl-phenyl rings in the molecular structure of the drugs (23). Drugs inhibit the hERG (human ether-a-go-go) subunit with which the channel conducts the main ventricular repolarization potassium current (*I*_Kr_) potential during phases 2–3 of the action potential (3, 24, 25). And hERG is regulated by cAMP and cAMP-dependent PKA. Vandetanib has been demonstrated to have values of hERG IC50 of 0.4 μM, and its metabolites are also active. In *in vivo* studies, a dose-dependent increase in QT prolongation has been demonstrated in dogs (3).

For instance, ibrutinib influences tumor cells by inhibiting Bruton tyrosine kinase (BTK); it also has off-target effects on Tec protein tyrosine kinase (TEC) (26). Both BTK and TEC transcripts have been demonstrated to express in cardiac tissue and at a higher expression when AF occurs rather than sinus rhythm. The PI3K-Akt pathway is regulated by BTK and TEC, and plays an important role in cardiac protection under conditions of stress (27).

Even if some drugs act on the same target or class of targets, it cannot be proved that these drugs have the effect of a prolonged QT interval. For example, sorafenib, vandetanib, and axitinib act on VEGFR, but only sorafenib and vandetanib will induce QT prolongation (23).

## Selected Representative Drugs

### Vandetanib

Vandetanib has the most significant prolongation of corrected QT interval according to Fridericia's formula (QTcF). It is an oral multitarget TKI drug, which inhibits VEGFR-2, EGFR, and the activity of tyrosine kinase. It was approved for the treatment of metastatic medullary thyroid cancer (MTC) by the FDA in 2011 (28).

In an early report, Ghatalia et al. initiated a meta-analysis about QTcF interval prolongation with VEGFR TKIs (29). They studied 13 clinical trials that included 4,204 patients with multi-tumor types who received 100 or 300 mg of vandetanib daily. The incidence of QT prolongation ranged from 0.3 to 23.9%, AF incidence ranged from 0.43 to 1.79%, and all-grade arrhythmia ranged from 0 to 1.69%. A high dose of vandetanib was associated with a high risk of QT prolongation by the authors. Then, under an approved dosage, phase II trial, multicenter, open-label study, 17 patients with metastatic or recurrent NSCLC with a RET rearrangement and against platinum-based doublet chemotherapy were treated with 300 mg of vandetanib once daily. A total of 6 out of 17 patients had grade 3 AEs, 2 were QT prolongation (11%) (30). In another phase III, double-blind, placebo-controlled clinical trial, 331 patients with advanced or metastatic MTC were randomized 2:1 to receive 300 mg of vandetanib daily orally (*n* = 231) or placebo (*n* = 100). During the treatment, most AEs could be well-managed by dose interruption or reduction, QT prolongation occurred in 14 patients in all grades (31).

Vandetanib is metabolized by cytochrome P450 enzyme (CYP) 3A4, which inhibits or promotes activity by many common drugs (32). In a phase I trial, healthy individuals received 200 mg of itraconazole daily and were given a single dose of 300 mg of vandetanib on day 1 and day 4. A slight increase (9%) was observed in the serum concentration of vandetanib (33). Other substrates of CYP3A4, such as ketoconazole and rifampicin should be considered in the drug combination. In addition, other drugs which may induce QT prolongation need to be considered in the drug combination.

### Ibrutinib

Ibrutinib has the highest incidence of AF. It is an oral irreversible small molecule inhibitor of Bruton's tyrosine kinase (BTK), which is inhibited by a covalent bond with the specific cysteine Cys-481 of BTK, thereby inhibiting the proliferation and survival of malignant B cells, as well as reducing their migration and substrate adhesion (34, 35). Through the inhibition of BTK, downstream signaling pathways (MAPK, PI3K, and NF-?B) and phosphorylation functions (PLCγ, ERK, and AKT) are influenced (35–37).

In a preclinical *in vitro* study, it was demonstrated that there were effects on hERG, but no specific risks for human cardiac issues, by the authors. In the *in vivo* safety study of dogs, ibrutinib may increase PR interval, decrease heart rate, and shorten heart rate-corrected QT interval (38). Besides, its use is associated with atrial toxicity. The possible mechanisms are still not entirely clear, but it has been demonstrated that AF is an off-target effect (39). Xiao et al., who used a mouse model and conducted chemo-proteomic analysis of cardiac lysates, found that C-terminal Src kinase (CSK) was the most likely target for ibrutinib-induced AF (40). Jiang et al. created a C57BI/6 mice model where an ibrutinib group received 25 mg/kg/d of ibrutinib and a control group was treated with hydroxypropy1-β-cyclodextrin for 4 weeks. Compared with the control group, the ibrutinib group displayed Ca^2+^ dysregulation in atrial myocytes, it increased spontaneous Ca^2+^ release, CaMKII level, phosphorylated CaMKII, and other related sites, and reduced sarcoplasmic Ca^2+^ capacity (41); both may lead to AF. Ibrutinib is an independent risk factor for the development of atrial arrhythmias, with an incidence of AF of more than 10–15% (42–44). Based on Alexandre's paper, ibrutinib is the most frequent anticancer drug to cause AF (45). In addition to straightforward arrhythmias, other potential effects of ibrutinib on ECG are little known. In early clinical trials, ~6–16% of participants had an increased risk of AF (46). A review of 16 studies showed that the incidence of ibrutinib-associated AF was 5.77 per 100 person-years (27). Fradley et al. enrolled 137 patients who were treated with ibrutinib, 21 pre- and post-ibrutinib ECG readings were obtained. Compared with the pre ibrutinib ECG, after administration, the ECG showed QT interval shortening from 446 to 437 ms, based on Bazett's formula (47). In another phase II clinical trial, a mean 7.5 ms shortening of the corrected QT interval was found after ibrutinib treatment (34). However, no significant QT effects were found in healthy subjects. Ibrutinib showed concentration-dependent mild shortening of the QT interval and PR prolongation, but seemed to have no significant clinical meaning (38).

Ibrutinib is metabolized by CYP3A4, therefore the coadministration of calcium channel blockers and other enzyme inhibitors should be fully considered. Besides, ibrutinib may increase the P-glycoprotein (P-gp) substrate level, such as digoxin and omeprazole, etc. (27, 48, 49).

### Ponatinib

Ponatinib is a third-generation TKI, which was designed by a computational and structure-based approach (50). It was created with a unique carbon-carbon triple bond linkage that overcomes the steric hindrance caused by the T315I mutant in CML or Ph^+^ acute lymphoblastic leukemia (ALL) (50, 51). The toxicity is mostly explained as a lack of selectivity, and on and off-target effects. Ponatinib inhibits over 60 kinases, including PDGFR, c-KIT, VEGFR, and EGFR. It also acts on perturbation of pro-survival signaling pathways, the AKT and ERK pathways impact cardiac function (52, 53). And in the Sharma et al. trial, they used human-induced pluripotent stem cell-derived cardiomyocytes (hiPSC-CMs) to evaluate the 21 approved TKIs, and demonstrated that ponatinib is the most toxic (54).

In a pre-clinical trial, ponatinib was not associated with cardiotoxicity. However, it exhibited a high occurrence of cardiac events in follow-up trials (11, 55). One year after being approved by the FDA, ponatinib was suspended because of safety concerns (56). At the dose of 50 mg/kg, the mean tumor volume decreased by 96%, so 45 mg once daily has been suggested, but the label cautions that an optimal dosage has not been identified (52, 57). In Sonnichsen's trial, 39 patients received ponatinib treatment at 30-, 45- and 60-mg dose levels, and the QTcF changes from baseline showed −10.9, −3.9, and −5.0 ms. Seventy-five patients in different dose levels were enrolled to evaluate the PK-PD effect, no significant correlation was found between drug exposure and QT changes (50). For the FDA CV events report for TKIs in 2020, 14.4% were found to be related to ponatinib (12). And in another clinical trial, 78 patients with CML were treated with ponatinib, the most common CAEs were arrhythmia (9%), higher than hypertension (7.7%) and myocardial infarction (3.8%) (58). In a ponatinib vs. imatinib phase III trial, 307 newly diagnosed CML patients were assigned to receive ponatinib (*n* = 155) or imatinib (*n* = 152). The results showed that no significant differences were observed in major molecule response at 12 months, but three serious AFs were observed in the ponatinib group, while no AFs occurred in the imatinib group (59).

Ponatinib is mostly metabolized by CYP3A4/5, but also by the substrates of CYP2C6 and CYP2C8. When co-administered with ketoconazole, the AUC_0−∞_, AUC_0−*t*_, and C_max_ indicated increased exposures to ponatinib of 78, 70, and 47%, respectively. So, a dose decreased to 30 from 45 mg daily could be considered when combined with strong CYP3A4 inhibitors (60, 61).

### Nilotinib

Nilotinib also has strong cardiotoxicity. Its ability to prolong QT interval and induce AF is just after that of vandetanib and ibrutinib. It is an orally administered small molecule TKI that was designed with a lipophilic structure which competitively binds to the inactive conformation of the ABL kinase domain and leads to a higher and faster molecule response than imatinib in patients (62, 63). Nilotinib is more selective than imatinib, and does not have an effect on Src, EGFR, and VEGF kinase at a concentration <3,000 nM (64–66). However, it targets PDGFRa, PDGFRb, c-kit, DDR, and colony-stimulating factor receptor 1, which is similar to imatinib (65).

Under the multi-target effect, nilotinib induce cardiotoxicity. In a preclinical safety study, nilotinib inhibited the hERG channel at an IC_50_ value of 0.13 μM (3), and exhibited the signs of QT prolongation in an isolated rabbit heart, but no toxicity in neonatal rat ventricular myocytes (67). There was no evidence that nilotinib had an effect on QTc in dogs at the dose up to 300 mg (3). In a response and safety phase I study, 33 patients with CML-BP were enrolled and received second-line nilotinib treatment. Thirteen patients (39%) achieved a hematologic response. As for arrhythmia, the QTcF increased by 5–15 ms in the study group, and one patient developed AF (grade = 2) (9). Then, in a phase II study, 280 patients with CML were enrolled, 6-month major cytogenetic responses were achieved in 48% of patients, and only 1% (3 of 280) had QTcF > 500 ms (62). In another phase II study, 44 patients with CML-AP or CML-CP were enrolled, and 6.1% displayed QT prolongation at all grades (68). A positive correlation was found between QT prolongation and nilotinib exposure in many trials (69–71). In patients, as C_max_ increased by 1,000 ng/ml, the QTcF also increased by 4.2 ms; an increase of 1,000 ng/ml in C_trough_ brought on an increase of 6.9 ms in QTcF (70, 71). Contrary to the earlier study, the 2020 FDA CV events report showed that QTc prolongation (any grade) induced by TKI was almost 28.8%, among these events 38.7% was due to nilotinib (12). It was significantly higher than early clinical trials. Alexandre et al. used the World Health Organization (WHO) individual case safety report database, vigibase, to identify the correlation between anticancer drugs and AF. As the results showed, 11,757 of 2,124,646 AF cases were associated with 176 anticancer drugs, and nilotinib accounted for 241 cases (2%) (45).

Nilotinib is also metabolized in the liver and is the competitive inhibitor of CYP3A4/5, CYP2C8, CYP2C9, CYP2D6, and uridine diphosphate glucuronosyltransferase 1A1 (UGT1A1) (72, 73). When co-administered with ketoconazole, the area under the curve (AUC) increased by 3-fold and C_max_ by 1.8-fold (74). Other drugs like rifampicin and esomeprazole reduced the plasma concentration to different degrees (72, 75).

### Dasatinib

Dasatinib seems to rarely cause QT interval prolongation events, and pooled safety data suggest that the overall risk for cardiotoxicity is minimal in dasatinib. But it still occurs in clinical use (76). Dasatinib is an effective BCR-ABL inhibitor in the treatment of CML and Philadelphia chromosome-positive acute lymphoblastic leukemia (Ph^+^ ALL) after relapse or resistance to imatinib (77, 78). It acts on the targets BCR-ABL, c-Kit, Src family kinases (SFKS), and PDGFR-α/β. By acting on BCR-ABL and the Src family, dasatinib inhibits the downstream PI3K signaling pathway (3, 79), which may induce cardiotoxicity.

The IC_50_ of the effect on hERG is 14.3 μM, which is safer than vandetanib in QT effects. In an *in vivo* study, no QT prolongation was found in monkeys using a body-weight dose strategy (10 or 70 mg/kg). In a clinical trial, 2,182 patients were treated with dasatinib, 21 displayed QT ≥ 500 ms, all patients had a mean increase from baseline of 3–5 ms (3). In another retrospective study, 115 cancer patients received dasatinib treatment, 41.7% of patients showed QT prolongation and the mean (SD) of pre- and post- therapeutic QT interval change was 30 ms (12).

Dasatinib influences CYP3A4 and CYP2C8 enzymes and the drug transporter P-gp. In drug-drug interaction, omeprazole, esomeprazole, and pantoprazole will inhibit the P-gp to decrease absorption and lead to higher exposure. Metoclopramide has an additive effect on dasatinib which will increase the incidence of QT interval (80, 81). In a phase I PK and drug interaction study, 17 patients were enrolled to determine whether the coadministration of ketoconazole affected the PK of dasatinib (82). Rhe result showed that ketoconazole led to an increase in dasatinib exposure and may correlate to an ~6 ms prolongation in QT interval.

The current study showed that QT prolongation occurred in nearly 30% of patients who received TKIs treatment, and 20% were high grade (7). Onco-cardiology is essential for cancer treatment because the incidence of cancer and cardiovascular diseases (CVD) is concentrated in middle-aged and senior patients. During the cancer treatment, it may aggravate CVD, and its side effects may lead to failure of cancer treatment (6). Other unexpected side effects include: Human epidermal growth factor receptor 2 (HER2) and trastuzumab are associated with left ventricular ejection fraction (LVEF) decrease and congestive heart failure (CHF) (83); anti-VEGF drugs were found to significantly increase the incidence of hypertension (84). However, arrhythmia could be induced by lots of targets.

These five TKIs all have obvious characteristics, mechanisms, aim targets, and AEs. Vandetanib and ibrutinib were found to have high incidences of arrhythmia but dasatinib was recommended not to be much concerns about cardiotoxicity. All of them exhibit an effect on QT interval and atria toxicity in clinical application. And, according to the FDA and WHO databases, the impact of some TKIs in QT and AF is much higher than in previous clinical trials (12, 45). Although QT interval prolongation has a lower mortality than hypertension or coronary heart disease, it is convenient as a monitoring strategy of CVD, and evaluation of QT interval changes can maximize the optimization of drug cessation or reduction. The main information of the selected representative drugs is summarized in Table 1.

**Table 1:** Target, effect on QT interval/AF, and metabolism of selected representative TKIs.

**Name**	**Vandetanib**	**Ibrutinib**	**Ponatinib**	**Nilotinib**	**Dasatinib**
Target	VEGFR-2; EGFR; RET	BTK; MAPK; PI3K	BCR-ABL; PDGFR; c-KIT; VEGFR; EGFR	BCR-ABL; PDGFR-α/β; c-kit; DDR	BCR-ABL; c-KIT; SFKS; PDGFR-α/β
Effect on QT interval	Prolong QT interval and with a positive drug exposure-dependent risk	Shorten QT interval	Prolong QT interval, no correlation was found between drug exposure and QT prolongation	Prolong QT interval with a positive correlation between exposure and risk	Rarely causes QT interval prolongation
Effect on AF	With a low incidence from 0.43 to 1.79%	Highest incidence of AF, nearly 10–15%	With a low incidence, about 1.29%	With a high incidence followed by ibrutinib	Rarely causes AF
Metabolized by	CYP3A4	CYP3A4	CYP3A4/5, CYP2C6, CYP2C8	CYP3A4	CYP3A4, CYP2C8

## Management

Arrhythmia induced by TKIs is easily monitored and can be an early warning of other serious CVD. For the agents with risk factors, routine monitoring methods such as ECG, blood pressure, electrolytes, and cardiac biomarkers are recommended during the course of treatment. In addition, collecting past medical history and physical function evaluation of patients are recommended to identify those at heightened risk for cardiovascular events. Once the arrhythmia occurs, beta-blockers and type I and III antiarrhythmic drugs are helpful for patients.

Primarily, CVD risk factors should be carefully evaluated before deciding to use TKIs. When patients are diagnosed with underlying diseases, like hypertension, coronary heart disease, diabetes, and other CVDs, they need to be carefully monitored based on their cardiac function. Baseline electrocardiograms (ECGs) should be obtained which will evaluate the risk of arrhythmia. Myoglobin (MYO), B-type natriuretic peptide (BNP), and other biochemical indexes should be obtained and corrected (85–87).

Second, electrolytes should be monitored, especially Na^+^, K^+^, and Ca^2+^ which have an influence on heart rhythm. Abnormal electrolytes will be a potential risk of arrhythmia and should be corrected immediately (87, 88).

Third, drug-drug interaction should be fully considered. Most TKIs are metabolized by CYP enzymes and transported by P-gp; other drugs influencing CYP enzymes and the competitive bond to P-gp should be fully considered during coadministration. And drugs that have been proved to prolong the QT interval or induce AF should be avoided (89, 90).

Fourth, reducing doses or stopping TKI treatment in time are of vital importance, especially when the QT interval is ≥500 ms, or the change of QT is > 60 ms compared with baseline. TKIs could be restarted when the QT is < 450 ms. If ventricular tachycardia, syncope, or other serious cardiovascular adverse reactions occurred again, the drugs should be stopped permanently (76, 91).

Fifth, after taking medicine, if a faint, headache, or irregular heartbeat occurs, healthcare should be provided immediately. The decision of heart rate or rhythm control should be patient-centered and symptom-oriented (87), beta-blockers may be the first choice for heart rate control, and type Ic and type III antiarrhythmic drugs are helpful for heart rhythm (87, 91).

## Conclusion

To conclude, we focused on the molecule mechanism, clinical outcome, coadministration, and countermeasures of TKI drugs in arrhythmia. There is commonality and variability coexistence in TKI class, studies showed that QT prolongation is the most significant in vandetanib, AF most occurs in ibrutinib, and nilotinib has a high incidence of QT prolongation and AF. Their actual incidence and life-threatening status are higher than preclinical trials, lots of them do not get a black box warning from the FDA. But CVD caused by antitumor drugs needs to be avoided during treatment. Therefore, early diagnosis, convenient monitoring measures, and appropriate treatment methods should be provided to patients. So, ECGs monitoring should be more widely used in cancer patients, existing guidelines should be more specific, more real-world clinical trials need to be done, and on-target and off-target toxicity should be completely understood in the future.

## Author Contributions

MC and FY collected data and wrote the paper. FY, JL, DY, SZ, and YY collected the literature and information. SJ, MD, and JL reviewed the paper. All authors read and approved the final manuscript.

## Funding

This work was supported by the Key Program of Harbin Medical University Cancer Hospital Haiyan Fund (no. JJZD2019-03) and the General Program of Harbin Medical University Cancer Hospital Haiyan Fund (no. JJMS2021-25).

## Conflict of Interest

The authors declare that the research was conducted in the absence of any commercial or financial relationships that could be construed as a potential conflict of interest.

## Publisher's Note

All claims expressed in this article are solely those of the authors and do not necessarily represent those of their affiliated organizations, or those of the publisher, the editors and the reviewers. Any product that may be evaluated in this article, or claim that may be made by its manufacturer, is not guaranteed or endorsed by the publisher.
